# Single-cell RNA sequencing identifies the properties of myelodysplastic syndrome stem cells

**DOI:** 10.1186/s12967-022-03709-9

**Published:** 2022-11-03

**Authors:** Yumei Liu, Haiyue Niu, Nan Song, Wei Zhang, Lijuan Li, Huaquan Wang, Rong Fu, Zonghong Shao

**Affiliations:** Department of Hematology, Tianjin Medical University General Hospital, 300052 Tianjin, China

## Letter to editor,

Myelodysplastic syndromes (MDS) are heterogeneous clonal diseases characterized by cytopenia caused by ineffective hematopoiesis and high risk of transformation into acute myeloid leukemia (AML). At present, the pathogenesis of MDS has not been elucidated. MDS is a group of stem cell diseases. The abnormal proliferation and blockade in differentiation of hematopoietic stem cells (HSCs) result in cytopenia and leukemic transformation. HSCs architectures in MDS can also predict therapeutic reaction [1]. Therefore, the high-resolution analysis of HSCs is of great significance. We investigated the properties of MDS stem cells by single-cell RNA sequencing (scRNA-seq). Lineage negative (Lin^−^) cells from bone marrow aspirates of 5 patients with MDS and 2 patients with secondary AML (sAML) were sorted out for scRNA-seq (Additional file 1: Additional methods, Additional file 2: Table S1). The scRNA-seq data of bone marrow mononuclear cells from 17 healthy donors (HDs) were downloaded from the Gene Expression Omnibus database (GSE120221) [2].

**Fig. 1:**
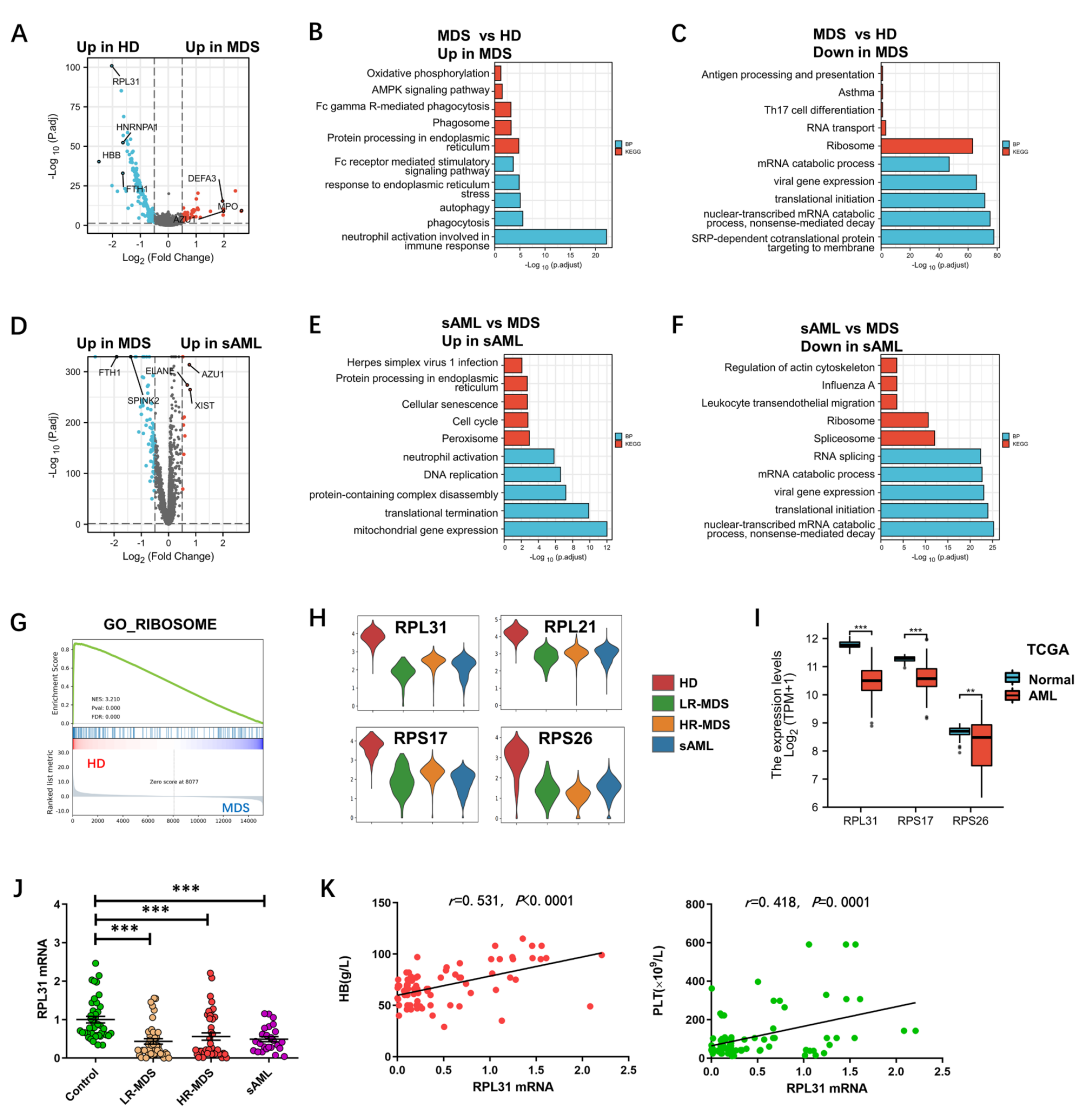
Analyses of aberrantly expressed genes and functional enrichment in myelodysplastic syndromes (MDS) stem cells by single-cell RNA sequencing **A** Volcano plot of differentially expressed genes (DEGs) in hematopoietic stem cells (HSCs)/multipotent progenitors (MPPs) between MDS patients and healthy donors (HDs). **B** Functional enrichment bar chart of up-regulated DEGs in HSCs/MPPs of MDS patients compared with HDs. Red: Kyoto Encyclopedia of Genes and Genomes (KEGG) gene sets, blue: Biological process (BP). **C** Functional enrichment bar chart of down-regulated DEGs in HSCs/MPPs of MDS patients compared with HDs. **D** Volcano plot of DEGs in HSCs/MPPs between secondary acute myeloid leukemia (sAML) and MDS patients. Red plot: up-regulated in sAML patients, blue plot: up-regulated in MDS patients. **E** Functional enrichment bar chart of up-regulated DEGs in HSCs/MPPs of sAML patients compared with MDS patients. **F** Functional enrichment bar chart of down-regulated DEGs in HSCs/MPPs of sAML patients compared with MDS patients. **G** Gene sets enrichment analysis (GSEA) showed decreased expression of ribosomal genes in HSCs/MPPs of MDS patients compared with HDs. **H** The violin plots of expression of ribosomal genes in HSCs/MPPs of HDs and patients with lower-risk MDS (LR-MDS), higher-risk MDS (HR-MDS) and sAML. **I** The Cancer Genome Atlas (TCGA) database analysis showed the expression of several ribosomal genes in AML patients. **J** Quantitative real-time PCR (qPCR) analyses of *RPL31* mRNA in CD34^+^ hematopoietic stem and progenitor cells from bone marrow of controls (n = 40), LR-MDS (n = 40), HR-MDS (n = 40) and sAML patients (n = 25). Significant difference is analyzed using analysis of variance. ***, P < 0.001. **K** Analyses of correlation between *RPL31* mRNA and hemoglobin (HB), platelet (PLT) in MDS patients (n = 80)

A total of 65,509 Lin^−^ hematopoietic cells were analyzed. After dimension reduction, clustering, and visualization, we identified HSC/multipotent progenitor (MPP) populations and conducted an in-depth analysis. We investigated the properties of MDS stem cells by analyzing the differentially expressed genes (DEGs) between patients and HDs. We found that genes associated with neutrophil granule, such as *MPO*, *AZU1*, *DEFA3*, had elevated expression in MDS patients compared with HDs (Fig. 1A and 1B). This is consistent with the tendency of predominantly myeloid differentiation trajectories of HSCs in MDS. We identified that the down-regulated DEGs in HSCs/MPPs were enriched in ribosome, translation, mRNA catabolic process, Th17 cell differentiation and antigen processing and presentation in MDS patients (Fig. 1C), which supported the opinion that MDS stem cells were in a relatively static state and had impaired immune function. The progression of MDS to AML usually bases on acquired mutations [3]. Our results suggested that abnormal proliferation, RNA metabolism and ribosome biogenesis also exist in MDS stem cells during leukemic transformation (Fig. 1D - F).

It is noteworthy that ribosomal genes were widely down-regulated in HSCs/MPPs of MDS/sAML patients compared with those of HDs (Fig. 1G and H), which suggested that abnormal ribosome biogenesis may be involved in the pathogenesis of MDS and leukemic transformation. The Cancer Genome Atlas (TCGA) database analysis also showed decreased expression of some ribosomal genes in AML (Fig. 1I). The results of quantitative real-time PCR verification showed that the expression of *RPL31* and *RPL21* mRNA in CD34^+^ cells of patients with LR-MDS (n = 40), HR-MDS (n = 40) and sAML (n = 25) were significantly lower than those of control (n = 40) (*P* < 0.001) (Fig. 1J, Additional file 3: Fig. S1A) and positively correlated with the levels of hemoglobin and platelet (Fig. 1K, Additional file 3: Fig. S1B).

Hematopoietic homeostasis depends on the balance between self-renewal and differentiation of HSCs. Increasing genome data showed that abnormal expression of some crucial regulatory genes may break the homeostasis and eventually lead to hematological malignant diseases. Our results revealed MDS stem cells have abnormal proliferation, ribosome biogenesis, RNA metabolism and impaired immune function. The expression of ribosomal genes was down-regulated in MDS stem cells. Abnormal ribosome biogenesis and regulation of translation was frequent in myeloid diseases. Gene mutations of ribosomal protein could lead to Diamond-Blackfan Anemia, Schwachman Diamond Syndrome, Congenital Dyskeratosis and some other diseases, all of which have defects in hematopoiesis. In addition, the dyserythropoiesis in MDS with isolated del(5q) is related to the loss of heterozygosity of *RPS14* gene. Haploinsufficiency of *RPS14* leads to activation of innate immune system and p53 pathway, excessive apoptosis of erythroblasts and megaloblastic anemia. Study by Saha et al.[4] showed that the expression of ribosomal genes in leukemia stem cells (LSCs) was down-regulated compared with normal HSCs, which was consistent with the results in our study. The biosynthesis of proteins in HSCs is lower than that in committed progenitor cells and differentiated cells [5]. LSCs may have similar mechanisms to survive and resist to chemotherapy. Therefore, abnormal ribosome biogenesis is an important feature during development and progression of MDS and may be also related to therapeutic resistance.

## Electronic supplementary material

Below is the link to the electronic supplementary material.

Supplementary Material 1

Supplementary Material 2

Supplementary Material 3

## Data Availability

The raw data reported in this study are deposited in the NCBI Sequence Read Archive under bioproject No.PRJNA720840.

## Abbreviations

DEG: differentially expressed gene
HD: healthy donor
HSC: hematopoietic stem cell
HR-MDS: higher-risk myelodysplastic syndrome
LSC: leukemia stem cell
Lin^−^: Lineage negative
LR-MDS: lower-risk myelodysplastic syndrome
MPP: multipotent progenitor
MDS: myelodysplastic syndrome
sAML: secondary acute myeloid leukemia
scRNA-seq: single-cell RNA sequencing
TCGA: The Cancer Genome Atlas
