# Supplementary material for: Single-cell RNA sequencing identifies the properties of myelodysplastic syndrome stem cells
Source: J Transl Med. 2022 Nov 3;20:499. doi: 10.1186/s12967-022-03709-9 (PMC9632008; doi:10.1186/s12967-022-03709-9)
Supplement: Supplementary file 1 — Supplementary Material 1 [file 12967_2022_3709_MOESM1_ESM.docx]

**Additional methods**

**Samples**

Lineage (CD2, CD3, CD11b, CD14, CD15, CD16, CD19, CD56, CD123 and CD235a) negative (Lin^-^) cells were sorted out by immunomagnetic beads (Miltenyi Biotec, Bergisch Gladbach, Germany) from bone marrow aspirates for Single-cell RNA sequencing (scRNA-seq).

**ScRNA-seq**

The protoplast suspension was loaded into Chromium microfluidic chips with 3’ v3 chemistry and barcoded with a 10X Chromium Controller (10X Genomics). RNA from the barcoded cells was subsequently reverse-transcribed and sequencing libraries were constructed with reagents from a Chromium Single Cell 3’ v3 reagent kit (10X Genomics). Sequencing was performed with Illumina NovaSeq 6000 (Illumina, San Diego, CA, USA).

**Data analysis**

Cell Ranger version 4.0.0 was used to demultiplex the raw FASTQ reads and align them to the human transcriptome (GRch38, provided by 10x Genomics) with the default parameters. The output of this pipeline contains a digital gene-barcode matrix for each sample. Then, all of the matrices were subjected to Scanpy version 1.6.0 for further data processing. Cells with more than 6,000 or less than 500 detected genes as well as those with a mitochondrial transcription ratio >20% were discarded. Cell doublets (occasional pairs of cells that are not dissociated during sample preparation) were identified using the scrublet tool and then discarded. Gene expression was performed using the SCTransform function provided by Seurat software. Next, harmonypy version 0.0.5 was employed to remove batch effects among samples. For clustering, Louvain algorithm was used, followed by uniform manifold approximation and projection (UMAP, version 0.4.6) visualization. The cell-specific markers were used for annotation.

**Differential expression analysis.** MAST version 1.14.0 was used to identify genes differentially expressed between conditions, which fit a hurdle model to the expression of each gene and performed zero-inflated regression analysis. The discrete and continuous coefficients of the model were retrieved, P values were computed by performing the likelihood ratio test, and q values were separately evaluated for each cell subset comparison using the Benjamini-Hochberg correction.

**Velocity analysis.** ScVelo version 0.2.2 was employed to infer transitional states for individual cells. Specifically, count matrices of unspliced and spliced abundance were obtained using the velocyto version 0.17 pipeline. Then, the matrices were input to scVelo for normalization, log-transformation and calculation of first- and second-order moments for each cell across its nearest neighbors. Next, the velocities for each cell were computed, and the velocity graph was constructed using the tl.velocity() function with the ‘stochastic’ mode. The latent time representing the internal clock of cells was obtained using the tl.latent_time() function with default parameters.

**Enrichment analysis of marker genes.** Enrichment analysis of Gene Ontology (GO) and Kyoto Encyclopedia of Genes and Genomes (KEGG) of differentially expressed genes (DEGs) was implemented by the cluster Profiler R package, in which gene length bias was corrected. GO terms with corrected p value less than 0.05 were considered significantly enriched by DEGs. Gene sets enrichment analysis (GSEA; http://software.broadinstitute.org/gsea) was used to test the enrichment of gene sets and pathways of detected genes.

**Trajectory inference.** Palantir was used to reconstruct a differentiation continuum of cells and observe the expression patterns of key genes.

**Quantitative real-time PCR (qPCR)**

After CD34^+^ hematopoietic stem and progenitor cells were sorted out by immunomagnetic beads (Miltenyi Biotec, Bergisch Gladbach, Germany) from bone marrow aspirates, total RNA was extracted by TRIzol (Thermo Fisher Scientific, Waltham, MA, USA) and reverse transcribed to cDNA. Finally, qPCR was done. The qPCR amplification conditions: 95 ℃ for 10 minutes, then 40 cycles of 95 ℃ for 15 seconds, 55 ℃ for 40 seconds, followed by a melting curve. The genes in our study have the same amplification conditions

**Statistical analyses**

Average log fold change (logFC) and q value were used for comparisons of gene expression in scRNA-seq data. Data of qPCR were analyzed by SPSS 25.0. Measurement data of normal distribution were compared with analysis of variance and expressed by means (± SEM). For non-normal distribution, nonparametric test was used, including Mann-Whitney test or Kruskal-Wallis test. Pearson and Spearman correlation were used for correlation analyses of normal distribution and non-normal distribution, respectively. P value< 0.05 was considered statistically significant.
