# Supplementary material for: Single-cell RNA sequencing identifies the properties of myelodysplastic syndrome stem cells
Source: J Transl Med. 2022 Nov 3;20:499. doi: 10.1186/s12967-022-03709-9 (PMC9632008; doi:10.1186/s12967-022-03709-9)
Supplement: Supplementary file 2 — Supplementary Material 2 [file 12967_2022_3709_MOESM2_ESM.docx]

**Table S1. Clinical characteristics of 7 patients with myelodysplastic syndromes and secondary acute myeloid leukemia.**

|  | Diagnosis | Sex  /Age | ANC(**×**10^9^/L) | HB  (g/L) | PLT(**×**10^9^/L) | Blast  (%) | karyotype | gene mutations |
| --- | --- | --- | --- | --- | --- | --- | --- | --- |
| Patient A | sAML | Female  /85 | 23.65 | 55 | 86 | 38.5 | normal | NA |
| Patient B | sAML | Male  /30 | 3.79 | 63 | 239 | 35.5 | normal | *RUNX1* |
| Patient C | EB-1 | Female  /66 | 1.82 | 82 | 232 | 5.0 | normal | NA |
| Patient D | isolated del(5q) | Male  /54 | 1.14 | 53 | 42 | 0.5 | del(5q) | *ASXL1* |
| Patient E | EB-2 | Female  /66 | 0.16 | 78 | 108 | 18.5 | normal | *ASXL1* |
| Patient F | EB-2 | Female  /47 | 3.98 | 57 | 80 | 12.5 | normal | NA |
| Patient G | RS-MLD | Female  /60 | 1.28 | 49 | 12 | 2.0 | normal | *DNMT3A* |

Note: sAML: secondary acute myeloid leukemia, ANC: absolute neutrophil count, HB: hemoglobin, PLT: platelet, RS-MLD: ring sideroblasts with multi-lineage dysplasia, EB: excess blasts, NA: not analyzed.
