# Supplementary material for: Single-cell RNA sequencing identifies the properties of myelodysplastic syndrome stem cells
Source: J Transl Med. 2022 Nov 3;20:499. doi: 10.1186/s12967-022-03709-9 (PMC9632008; doi:10.1186/s12967-022-03709-9)
Supplement: Supplementary file 3 — Supplementary Material 3 [file 12967_2022_3709_MOESM3_ESM.docx]

**Fig. S1**

**
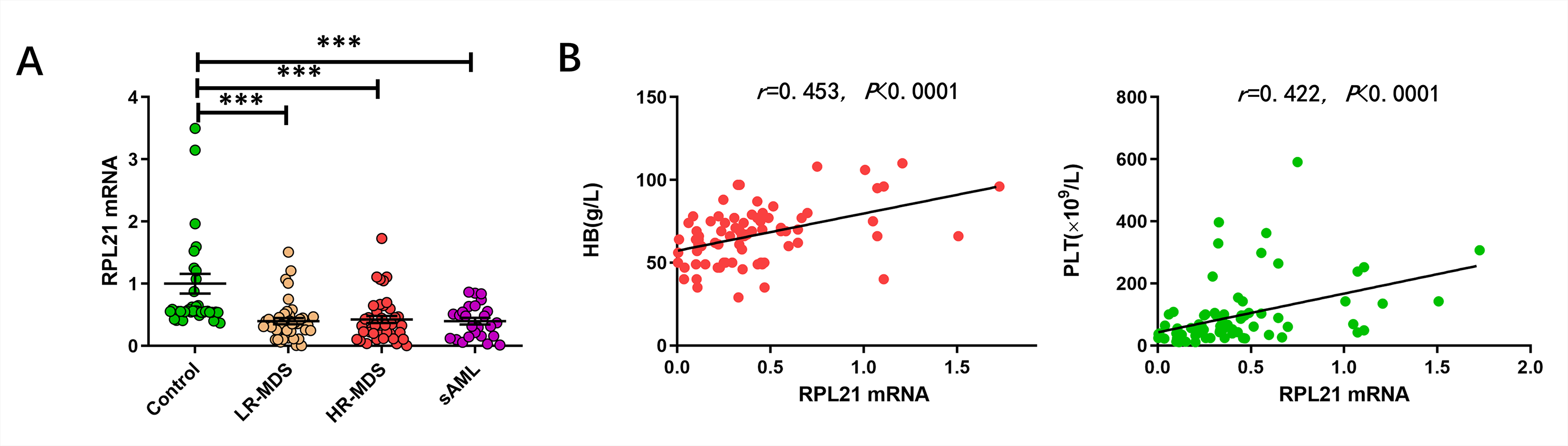
**

**Fig. S1. QPCR verification of ribosomal genes in CD34^+^ hematopoietic cells of controls, MDS and sAML patients..** **A** QPCR analyses of *RPL21* mRNA in CD34^+^ cells from bone marrow of controls (n=40), LR-MDS (n=40), HR-MDS (n=40) and sAML patients (n=25). Significant difference is analyzed using analysis of variance. ***, P <0.001. **B** Analyses of correlation between *RPL21* mRNA and hemoglobin (HB), platelet (PLT) in MDS patients (n=80).
